# Supplementary material for: Study of heavy metal resistance genes in Escherichia coli isolates from a marine ecosystem with a history of environmental pollution (arsenic, cadmium, copper, and mercury)
Source: PLoS One. 2023 Nov 16;18(11):e0294565. doi: 10.1371/journal.pone.0294565 (PMC10653420; doi:10.1371/journal.pone.0294565)
Supplement: S2 Table — (DOCX) [file pone.0294565.s002.docx]

**Supplementary Table 2. Co-resistance and linkage of heavy metal resistance genes (HMRGs), antibiotic resistance genes (ARGs), and plasmids in *E. coli* isolates by Salish Sea region and isolate sampling source (N=25).**

| Isolate Project ID | Isolate Sampling Source | Acquired HMRGs^a,e^  (start-stop positions in contig) | Acquired ARGs^a,e^  (start-stop positions in contig) | Resistance Phenotype by Antibiotic | Plasmid Replicons^a,e^  (start-stop positions in contig) |
| --- | --- | --- | --- | --- | --- |
| North Salish Sea | | | |  |  |
| 339942-001-501 | Drayton Harbor | *arsB*^d*^ (1..429)*, arsC* (1..141)^*^*, arsR*^d*^ (1..117)*, bhsA/ycfR/comC* (1..85)*, comR/ycfQ*^*^ (22..231)*, cusR/ylcA*^*^ (1..227)*, cusS*^*^ (1..479)*, cutA*^*^ (1..112)*, cutC*^*^ (1..248)*, cutF/nlpE*^*^ (1..236)*, dsbA*^*^ (1..208)*, dsbB*^*^ (1..176)*, dsbC*^*^ (1..236)*, robA*^*^ (1..289)*, ygiW*^*^ (1..130)*, zinT/yodA*^*^ (1..125) | *aadA2* (4352..5143),  *dfrA12* (3447..3944),  *qnrB19*^*^ (414..1058),  *sul3* (6314..7105),  *tet*(A)^*^ (108..1382) | Minocycline, Trimethoprim/  Sulfamethoxazole | IncX1 (16253..16629) |
| 339942-002-506 | Drayton Harbor | *arsB*^d*^ (1..429)*, arsC*^*^ (1..141)*, arsR*^d*^ (1..117)*, bhsA/ycfR/comC* (1..85)*, comR/ycfQ*^*^ (22..231)*, cusR/ylcA*^*^ (1..227)*, cusS*^*^ (1..479)*, cutA* (1..112)*, cutC*^*^ (1..248)*, cutF/nlpE*^*^ (1..236)*, dsbA*^*^ (1..208)*, dsbB*^*^ (1..176)*, dsbC*^*^ (1..236)*, robA*^*^ (1..289)*, ygiW*^*^ (1..130)*, zinT/yodA*^*^ (9..223) | *aadA2* (4559..5350),  *bla*_CTX-M-15_ (19232..20107),  *dfrA12* (3654..4151),  *qnrB19*^*^ (415..1059),  *qnrS1* (24748..25404),  *sul3* (6521..7312),  *tet*(A)^*^ (108..1382) | Aztreonam, Cefotaxime, Doxycycline, Trimethoprim/  Sulfamethoxazole, Ciprofloxacin (Intermediate) | IncFIB(AP001918) (7912..8593),  IncX1 (26475..26851) |
| 339942-003-511 | Drayton Harbor | *arsB*^d^ (1..429)*, arsC* (1..141)*, arsR*^d^ (1..117)*, bhsA/ycfR/comC* (1..85)*, comR/ycfQ* (22..231)*, cusR/ylcA* (1..227)*, cusS* (1..479)*, cutA* (1..112)*, cutC* (1..248)*, cutF/nlpE* (1..236)*, dsbA* (1..208)*, dsbB* (1..176)*, dsbC* (1..236)*, robA* (1..289)*, ygiW* (1..130)*, zinT/yodA* (1..216) | *aadA1* (69458..70249),  *aac(3)-Via* (68392..69294),  *aph(3'')-Ib* (42585..43388),  *aph(6)-Id* (41749..42585),  *bla*_SHV-12_ (23026..23886),  *sul1* (61553..62392),  *tet*(A) (47837..49036) | Cefotaxime, Doxycycline (Intermediate), Gentamicin (Intermediate) | IncFIB(AP001918) (8936..9617) |
| 351565-001-1202 | Portage Bay | *arsB*^d^ (1..429)*, arsC* (1..141)*, arsR*^d^ (1..117)*, bhsA/ycfR/comC* (1..85)*, comR/ycfQ* (22..231)*, cusR/ylcA* (1..227)*, cusS*^*^ (1..480)*, cutA* (1..112)*, cutC* (1..248)*, cutF/nlpE* (1..236)*, dsbA* (1..208)*, dsbB* (1..176)*, dsbC* (1..236)*, robA* (1..289)*, ygiW* (1..130)*, zinT/yodA* (9..223) | *aadA5* (2579..3367),  *aph(3'')-Ib* (1179..1982),  *aph(6)-Id* (1982..2818),  *bla*_TEM-1B_ (4084..4944),  *catA1*^*^ (351..1010),  *dfrA17* (1975..2448),  *mph(A)* (10064..10969),  *sul1* (3914..4753),  *sul2*^*^ (303..1118),  *tet*(A) (4079..5353),  *tet*(B) (4300..5505) | Ciprofloxacin, Doxycycline, Levofloxacin, Minocycline, Trimethoprim/  Sulfamethoxazole | IncFIB(AP001918) (706..1387),  IncQ1^b*^ (135..663) |
| Central Salish Sea | | | |  |  |
| 339940-002-477 | Yukon Harbor^c^ | *arsB*^d^ (1..429)*, arsC* (1..141)*, arsR*^d^ (1..117)*, bhsA/ycfR/comC* (1..85)*, comR/ycfQ* (22..231)*, cusR/ylcA* (1..227)*, cusS* (1..479)*, cutA* (1..112)*, cutC* (1..248)*, cutF/nlpE* (1..53; 1..236)*, dsbA* (1..208)*, dsbB* (1..176)*, dsbC* (1..236)*, robA* (1..289)*, ygiW* (1..23; 1..130)*, zinT/yodA* (9..223) | *bla*_CMY-2_ (24580..25725) | Cefotaxime, Ceftazidime, Ticarcillin/ Clavulanic Acid (Intermediate) | IncI1-Iα (16223..16364) |
| 344914-013-1036 | Liberty Bay^c^ | *arsB*^d^ (1..429; 1..32)*, arsC* (1..141)*, arsR*^d^ (1..117)*, bhsA/ycfR/comC* (1..85)*, comR/ycfQ* (22..231)*, cusR/ylcA* (1..227)*, cusS* (1..479)*, cutA* (1..112)*, cutC* (1..248)*, cutF/nlpE* (1..53; 1..236)*, dsbA* (1..208)*, dsbB* (1..176)*, dsbC* (1..236)*, robA* (1..289)*, ygiW* (1..130)*, zinT/yodA* (1..216) | *aph(3'')-Ib* (2507..3310),  *aph(6)-Id* (3310..4146),  *tet*(B) (1429..2634) | Doxycycline, Minocycline (Intermediate) | IncFIB(AP001918) (20872..21553) |
| Fresh Water | | | |  |  |
| GG 14-5 Cef | Golden Gardens | *arsB*^d^ (1..429)*, arsC* (1..141)*, arsR*^d^ (1..117)*, bhsA/ycfR/comC* (1..85)*, comR/ycfQ* (22..231)*, cusR/ylcA*^*^ (1..227)*, cusS*^*^ (1..479)*, cutA* (1..112)*, cutC*^*^ (1..248)*, cutF/nlpE*^*^ (1..236)*, dsbA*^*^ (1..208)*, dsbB* (1..176)*, dsbC*^*^ (1..236)*, robA*^*^ (1..289)*, ygiW* (1..130)*, zinT/yodA*^*^ (9..223) | *bla*_CTX-M-15_ (95789..96664),  *mph(A)*^*^ (177..1082),  *qnrS1* (101305..101961) | Aztreonam, Cefotaxime, Ceftazidime (Intermediate), Cefepime | -- |
| GG 14-6 Cef | Golden Gardens | *arsB*^d^ (1..429)*, arsC* (1..141)*, arsR*^d^ (1..117)*, bhsA/ycfR/comC* (1..85)*, comR/ycfQ*^*^ (27..236)*, cusR/ylcA*^*^ (1..227)*, cusS*^*^ (1..479)*, cutA* (1..112)*, cutC*^*^ (1..248)*, cutF/nlpE*^*^ (1..236)*, dsbA* (1..208)*, dsbB* (1..176)*, dsbC*^*^ (1..236)*, robA*^*^ (1..289)*, ygiW* (1..130)*, zinT/yodA*^*^ (1..216) | *aadA2* (7771..8562),  *bla*_CTX-M-15_ (11916..12791),  *catA1* (1563..2222),  *dfrA12* (8970..9467),  *mph(A)*^*^ (211..1116),  *qepA4* (2906..4441),  *sul1* (6427..7266),  *tet*(B) (913..2118), | Aztreonam, Cefepime, Cefotaxime, Ceftazidime, Ciprofloxacin, Doxycycline, Levofloxacin, Minocycline, Ticarcillin/  Clavulanic Acid, Trimethoprim/  Sulfamethoxazole | IncFIA (9297..9684) |
| PCB4Cef | Piper's Creek | *arsB*^d^ (1..429)*, arsC* (1..141)*, arsR*^d^ (1..117)*, bhsA/ycfR/comC* (1..85)*, comR/ycfQ* (22..231)*, cusR/ylcA* (1..227)*, cusS* (1..479)*, cutA* (1..112)*, cutC* (1..248)*, cutF/nlpE* (1..236)*, dsbA* (1..208)*, dsbB* (1..176)*, dsbC* (1..236)*, robA* (1..289)*, ygiW* (1..130)*, zinT/yodA* (1..216) | *bla*_CMY-2_ (42209..43354) | Ampicillin, Amoxicillin/ Clavulanic Acid, Ceftriaxone, Aztreonam, Ceftazidime, Ticarcillin/ Clavulanic Acid (Intermediate) | IncI1-Iα (1711..1852),  IncX1 (32631..32258) |
| Harbor Seal | | | |  |  |
| EPA Dock G Cip 1#5 | Manchester^c^ | *arsB*^d^ (1..429)*, arsC* (1..141)*, arsR*^d^ (1..117)*, bhsA/ycfR/comC* (1..85)*, comR/ycfQ* (22..231)*, cusR/ylcA* (1..227)*, cusS*^*^ (1..480)*, cutA* (1..112)*, cutC* (1..248)*, cutF/nlpE* (1..236)*, dsbA* (1..208)*, dsbB* (1..176)*, dsbC* (1..236)*, robA* (1..289)*, ygiW* (1..130)*, zinT/yodA* (9..223) | *aph(3'')-Ib* (1179..1982),  *aph(6)-Id*^*^ (1982..2818),  *catA1*^*^ (363..1022),  *sul2*^*^ (303..1118),  *tet*(A) (4079..5353) | Ciprofloxacin, Doxycycline (Intermediate), Levofloxacin | IncQ1^b*^ (136..664) |
| EPA Dock G#1 | Manchester^c^ | *arsB*^d^ (1..429)*, arsC* (1..141)*, arsR*^d^ (1..117)*, bhsA/ycfR/comC* (1..85)*, comR/ycfQ* (22..231)*, cusR/ylcA* (1..227)*, cusS* (1..479)*, cutA* (1..112)*, cutC* (1..248)*, cutF/nlpE* (1..236)*, dsbA* (1..208)*, dsbB* (1..176)*, dsbC* (1..236)*, robA* (1..288)*, ygiW* (1..130)*, zinT/yodA* (1..216) | *aadA5* (15231..16019),  *dfrA17* (14627..15100),  *sul2* (3947..4762),  *tet*(B) (694..1899) | Doxycycline, Trimethoprim/  Sulfamethoxazole, Minocycline (Intermediate) | -- |
| HASE 6 CEF | Grays Harbor | *arsB*^d^ (1..429)*, arsC* (1..141)*, arsR*^d^ (1..117)*, bhsA/ycfR/comC* (1..85)*, comR/ycfQ* (1..210)*, cusR/ylcA* (1..227)*, cusS* (1..479)*, cutA* (1..112)*, cutC* (1..248)*, cutF/nlpE* (1..236)*, dsbA* (1..208)*, dsbB* (1..176)*, dsbC* (1..236)*, pcoA* (1..605), *pcoB* (3..298), *pcoC* (1..126), *pcoD* (1..309)*, robA* (1..289)*, ygiW* (1..130)*, zinT/yodA* (1..216) | *bla*_CMY-2_ (3657..4802) | Ampicillin, Amoxicillin/ Clavulanic Acid, Ceftriaxone, Aztreonam, Cefotaxime, Ceftazidime, Ticarcillin/ Clavulanic Acid (Intermediate) | IncFIB(AP001918) (6766..7447),  IncI1-Iα (1999..2140) |
| SKMMR2020-01-025 Fecal #1 | Seattle | *arsB*^d^ (1..429)*, arsC* (1..141)*, arsR*^d^ (1..117)*, bhsA/ycfR/comC* (1..85)*, comR/ycfQ* (22..231)*, cusR/ylcA* (1..227)*, cusS*^*^ (1..480)*, cutA* (1..112)*, cutC* (1..248)*, cutF/nlpE* (1..236)*, dsbA* (1..208)*, dsbB* (1..176)*, dsbC* (1..236)*, robA* (1..289)*, ygiW* (1..130)*, zinT/yodA* (9..223) | *aph(3'')-Ib* (1179..1982),  *aph(6)-Id* (1982..2818)  *catA1*^*^ (363..1022),  *sul2*^*^ (303..1118),  *tet*(A) (4079..5353) | Ciprofloxacin, Levofloxacin | IncQ1^b*^ (136..664) |
| SKMMR2020-01-025 Gut #1 | Seattle | *arsB*^d^ (1..429)*, arsC* (1..141)*, arsR*^d^ (1..117)*, bhsA/ycfR/comC* (1..85)*, comR/ycfQ* (22..231)*, cusR/ylcA* (1..227)*, cusS* (1..479)*, cutA* (1..112)*, cutC* (1..248)*, cutF/nlpE* (1..236)*, dsbA* (1..208)*, dsbB* (1..176)*, dsbC* (1..236)*, robA* (1..289)*, ygiW* (1..130)*, zinT/yodA* (9..223) | *dfrA5* (34432..34905) | Trimethoprim/  Sulfamethoxazole | IncFIB(AP001918) (14815..15496),  IncFII (26061..26321) |
| SSW080719 (AN0077) | Alki Beach | *arsB*^d^ (1..429)*, arsC* (1..141)*, arsR*^d^ (1..117)*, bhsA/ycfR/comC* (1..85)*, comR/ycfQ* (1..210)*, cusR/ylcA* (1..227)*, cusS* (1..480)*, cutA* (1..112)*, cutC* (1..248)*, cutF/nlpE* (1..235)*, dsbA* (1..208)*, dsbB* (1..176)*, dsbC* (1..236)*, pcoA* (1..605), *pcoB* (3..298), *pcoC* (1..126), *pcoD* (1..309)*, robA* (1..289)*, ygiW* (1..130)*, zinT/yodA* (1..216) | *aph(3'')-Ib* (3844..4647),  *aph(6)-Id* (4647..5483),  *sul2* (1358..2173),  *tet*(B) (4300..5505) | Doxycycline | IncFIB(AP001918) (14859..15540) |
| SSW082919 (AN0092) | Lincoln Park | *arsB*^d^ (1..429)*, arsC* (1..141)*, arsR*^d^ (1..117)*, bhsA/ycfR/comC* (1..85)*, comR/ycfQ* (1..210)*, cusR/ylcA* (1..227)*, cusS* (1..480)*, cutA* (1..112)*, cutC* (1..248)*, cutF/nlpE* (1..235)*, dsbA* (1..208)*, dsbB* (1..176)*, dsbC* (1..236)*, pcoA* (1..605), *pcoB* (3..298), *pcoC* (1..126), *pcoD* (1..309)*, robA* (1..289)*, ygiW* (1..130)*, zinT/yodA* (1..216) | *aph(3'')-Ib* (3844..4647),  *aph(6)-Id* (4647..5483),  *sul2* (881..1696),  *tet*(B) (4217..5422) | Doxycycline | IncFIB(AP001918) (14859..15540) |
| WDFW2019-154 (AN0107) | Bremerton^c^ | *arsC* (1..141)*, bhsA/ycfR/comC* (1..85)*, comR/ycfQ* (1..210)*, cusR/ylcA* (1..227)*, cusS* (1..480)*, cutA* (1..112)*, cutC* (1..248)*, cutF/nlpE* (1..236)*, dsbA* (1..208)*, dsbB* (1..176)*, dsbC* (1..236)*,*  *robA* (1..289)*, ygiW* (1..130)*, zinT/yodA* (1..216) | *aac(3)-IId* (179968..180828),  *aadA2* (2627..3418),  *bla*_TEM-1B_ (174136..174996),  *dfrA12* (1722..2219),  *mph(A)* (10073..10978),  *sul1*^*^(3923..4762) | Amoxicillin, Gentamicin, Trimethoprim/  Sulfamethoxazole | Col156^*^ (3779..3932),  IncFII(29) (40167..40425) |
| River Otter | | | |  |  |
| BR1E | Black River^c^ | *arsB*^d*^ (1..429)*, arsC* (1..141)*, arsR*^d^ (1..117)*, bhsA/ycfR/comC* (1..85)*, comR/ycfQ*^*^ (22..231)*, cusR/ylcA*^*^ (1..227)*, cusS*^*^ (1..479)*, cutA* (1..112)*, cutC*^*^ (1..248)*, cutF/nlpE*^*^ (1..236)*, dsbA*^*^ (1..208)*, dsbB* (1..176)*, dsbC*^*^ (1..236)*, pcoA*^*^ (1..605), *pcoB*^*^ (3..298), *pcoC* (1..126), *pcoD*^*^ (1..309)*, robA*^*^ (1..289)*, ygiW* (1..130)*, zinT/yodA*^*^ (9..223) | *aac(3)-IV*^*^ (192..968),  *aadA1* (39657..40445),  *aph(3'')-Ib*^*^ (319..1122),  *aph(4)-Ia* (1197..2222),  *aph(6)-Id* (1122..1958),  *bla*_TEM-1B_^*^ (289..1149),  *lnu(F)* (41078..41899),  *tet*(B) (1429..2634) | Doxycycline, Gentamicin, Tobramycin, Minocycline (Intermediate) | IncI1-Iα (20572..20713) |
| BR1F | Black River^c^ | *arsB*^d*^ (1..429)*, arsC* (1..141)*, arsR*^d^ (1..117)*, bhsA/ycfR/comC* (1..85)*, comR/ycfQ*^*^ (22..231)*, cusR/ylcA*^*^ (1..227)*, cusS*^*^ (1..479)*, cutA* (1..112)*, cutC*^*^ (1..248)*, cutF/nlpE*^*^ (1..236)*, dsbA*^*^ (1..208)*, dsbB* (1..176)*, dsbC*^*^ (1..236)*, pcoA*^*^ (1..605), *pcoB*^*^ (3..298), *pcoC* (1..126), *pcoD*^*^ (1..309)*, robA*^*^ (1..289)*, ygiW* (1..130)*, zinT/yodA*^*^ (9..223) | *aac(3)-IV*^*^ (192..968),  *aadA1* (39657..40445),  *aph(3'')-Ib*^*^ (319..1122),  *aph(4)-Ia* (1197..2222),  *aph(6)-Id* (1122..1958),  *bla*_TEM-1B_^*^ (289..1149),  *lnu(F)* (41078..41899),  *tet*(B) (2310..3515) | Ampicillin, Gentamicin, Tetracycline, Minocycline | IncI1-Iα (20572..20713) |
| CWG3I | Cottonwood Grove Park^c^ | *arsB*^d^ (1..429)*, arsC* (1..141)*, arsR*^d^ (1..117)*, bhsA/ycfR/comC* (1..85)*, comR/ycfQ* (22..231)*, cusR/ylcA* (1..227)*, cusS* (1..479)*, cutA* (1..112)*, cutC* (1..248)*, cutF/nlpE* (1..236)*, dsbA* (1..208)*, dsbB* (1..176)*, dsbC* (1..236)*, robA* (1..289)*, ygiW* (1..130)*, zinT/yodA* (9..223) | *tet*(B) (1448..2653) | Cefotaxime (Intermediate), Tetracycline, Minocycline (Intermediate), Sulfisoxazole (Intermediate) | -- |
| CWG3J | Cottonwood Grove Park^c^ | *arsB*^d^ (1..429)*, arsC* (1..141)*, arsR*^d^ (1..117)*, bhsA/ycfR/comC* (1..85)*, comR/ycfQ* (22..231)*, cusR/ylcA* (1..227)*, cusS* (1..479)*, cutA* (1..112)*, cutC* (1..248)*, cutF/nlpE* (1..16; 1..236)*, dsbA* (1..208)*, dsbB* (1..176)*, dsbC* (1..236)*, robA* (1..289)*, ygiW* (1..130)*, zinT/yodA* (9..223) | *aadA1* (11340..12131),  *aadA2b* (13745..14524),  *cmlA1* (12224..13483),  *sul3* (8867..9658),  *tet*(A) (2141..3340) | Chloramphenicol, Tetracycline, Sulfisoxazole, Minocycline (Intermediate) | IncI1-Iα (3630..3771),  p0111 (25014..25898) |
| GRNRA2B | Green River Natural Resource Area^c^ | *arsC* (1..141)*, bhsA/ycfR/comC* (1..85)*, comR/ycfQ* (1..210)*, cusR/ylcA* (1..227)*, cusS* (1..480)*, cutA* (1..112)*, cutC* (1..248)*, cutF/nlpE* (1..236)*, dsbA* (1..208)*, dsbB* (1..176)*, dsbC* (1..236)*,*  *robA* (1..289)*, ygiW* (1..130)*, zinT/yodA* (1..216) | *bla*_TEM-1C_ (165149..166009) | Ampicillin, Imipenem (Intermediate), Kanamycin (Intermediate), Sulfisoxazole (Intermediate) | -- |
| GRNRA2E | Green River Natural Resource Area^c^ | *arsC* (1..141)*, bhsA/ycfR/comC* (1..85)*, comR/ycfQ* (1..210)*, cusR/ylcA* (1..227)*, cusS* (1..480)*, cutA* (1..112), *cutC* (1..248)*, cutF/nlpE* (1..235)*, dsbA* (1..208)*, dsbB* (1..176)*, dsbC* (1..236)*,*  *robA* (1..289)*, ygiW* (1..130)*, zinT/yodA* (1..216) | *aac(2')-Iia* (340306..341088) | Cefotaxime, Sulfisoxazole, Ampicillin (Intermediate), Imipenem (Intermediate), Meropenem (Intermediate), Amikacin (Intermediate) | -- |
| HAM5E | Hamm Creek^c^ | *arsB*^d*^ (1..429)*, arsC* (1..141)*, arsD*^d^ (1..120)*, arsR*^d^ (1..85)*, bhsA/ycfR/comC* (1..85)*, comR/ycfQ*^*^ (22..231)*, cusR/ylcA* (1..227)*, cusS*^*^ (1..479)*, cutA* (1..112)*, cutC*^*^ (1..248)*, cutF/nlpE*^*^ (1..236)*, dsbA*^*^ (1..208)*, dsbB* (1..176)*, dsbC*^*^ (1..236)*, robA*^*^ (1..289)*, ygiW* (1..130)*, zinT/yodA*^*^ (9..223) | *aadA5* (391..1179),  *aph(3'')-Ib* (994..1797),  *aph(6)-Id** (158..994),  *bla*_TEM-1B_ (676..1536),  *catA1** (239..898),  *dfrA17* (1310..1783),  *qnrB19** (1..400; 1..552),  *sul2* (1858..2673),  *tet*(B) (2523..3728) | Ampicillin, Trimethoprim/  Sulfamethoxazole, Tetracycline, Minocycline, Sulfisoxazole | Col(pHAD28)^b*^ (1..118, 35..164, 75..205, 93..223, 511..386, 521..612), 1951..2081),  Col156 (3772..3913),  Col8282 (2247..2453),  IncFII(29) (30056..30314),  IncQ1 (4012..4540)**^b^** |
| HAM6D | Hamm Creek^c^ | *arsB*^d^ (1..429)*, arsC* (1..141)*, arsR*^d^ (1..117)*, bhsA/ycfR/comC* (1..85)*, comR/ycfQ* (22..231)*, cusR/ylcA* (1..227)*, cusS* (1..480)*, cutA* (1..112)*, cutC* (1..248)*, cutF/nlpE* (1..236)*, dsbA* (1..208)*, dsbB* (1..176)*, dsbC* (1..236)*, pcoA*^d^ (1..605), *pcoB*^d^ (3..298), *pcoC*^d^ (1..126), *pcoD*^d^ (1..177)*, robA* (1..289)*, ygiW* (1..130)*, zinT/yodA* (9..223) | *aph(3'')-Ib* (1601..2404),  *aph(6)-Id* (2404..3240),  *bla*_TEM-1B_ (1677..2537),  *tet*(B) (983..2188) | Ampicillin, Trimethoprim/  Sulfamethoxazole, Tetracycline | IncI1-Iα (16046..16187),  IncR (7687..7937) |

^a^Minimum 90% identity threshold between the best matching resistance gene in the database and the corresponding sequence in the genome and 100% minimum alignment length, unless otherwise indicated.

^b^Minimum 90% identity threshold between the best matching resistance gene in the database and the corresponding sequence in the genome and 60% minimum alignment length.

^c^Sources located near United States Environmental Protection Agency designated Superfund sites on the United States Environmental Protection Agency National Priorities List.

^d^HMRG was not found in all study isolates.

^*^There was overlap in the sequence with the indicated HMRGs, ARGs, and/or plasmids.
