# Supplementary figures and images for: Study of heavy metal resistance genes in Escherichia coli isolates from a marine ecosystem with a history of environmental pollution (arsenic, cadmium, copper, and mercury)
Source: PLoS One. 2023 Nov 16;18(11):e0294565. doi: 10.1371/journal.pone.0294565 (PMC10653420; doi:10.1371/journal.pone.0294565)

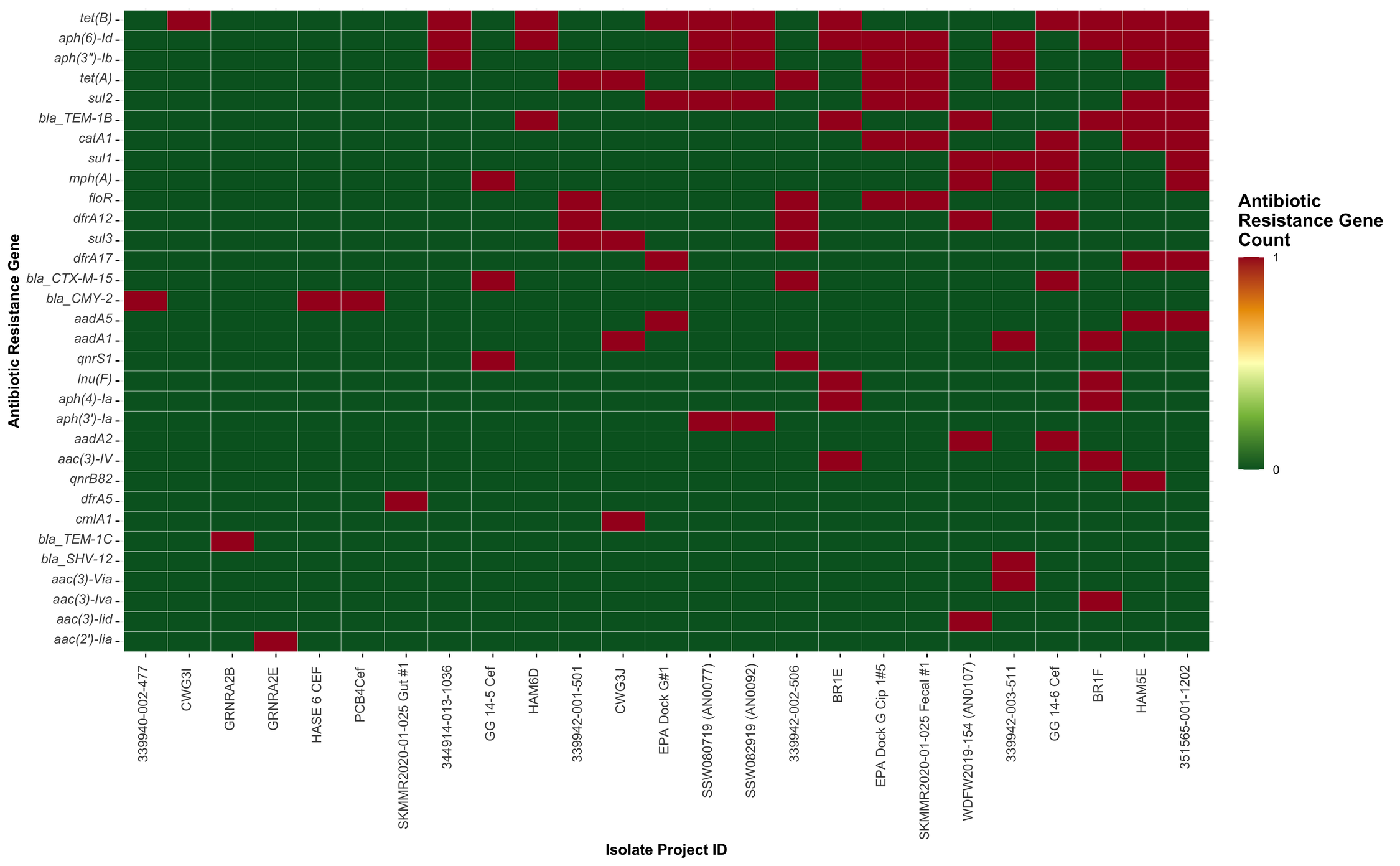

Supplement: S1 Fig — (TIF) [file pone.0294565.s004.tif]

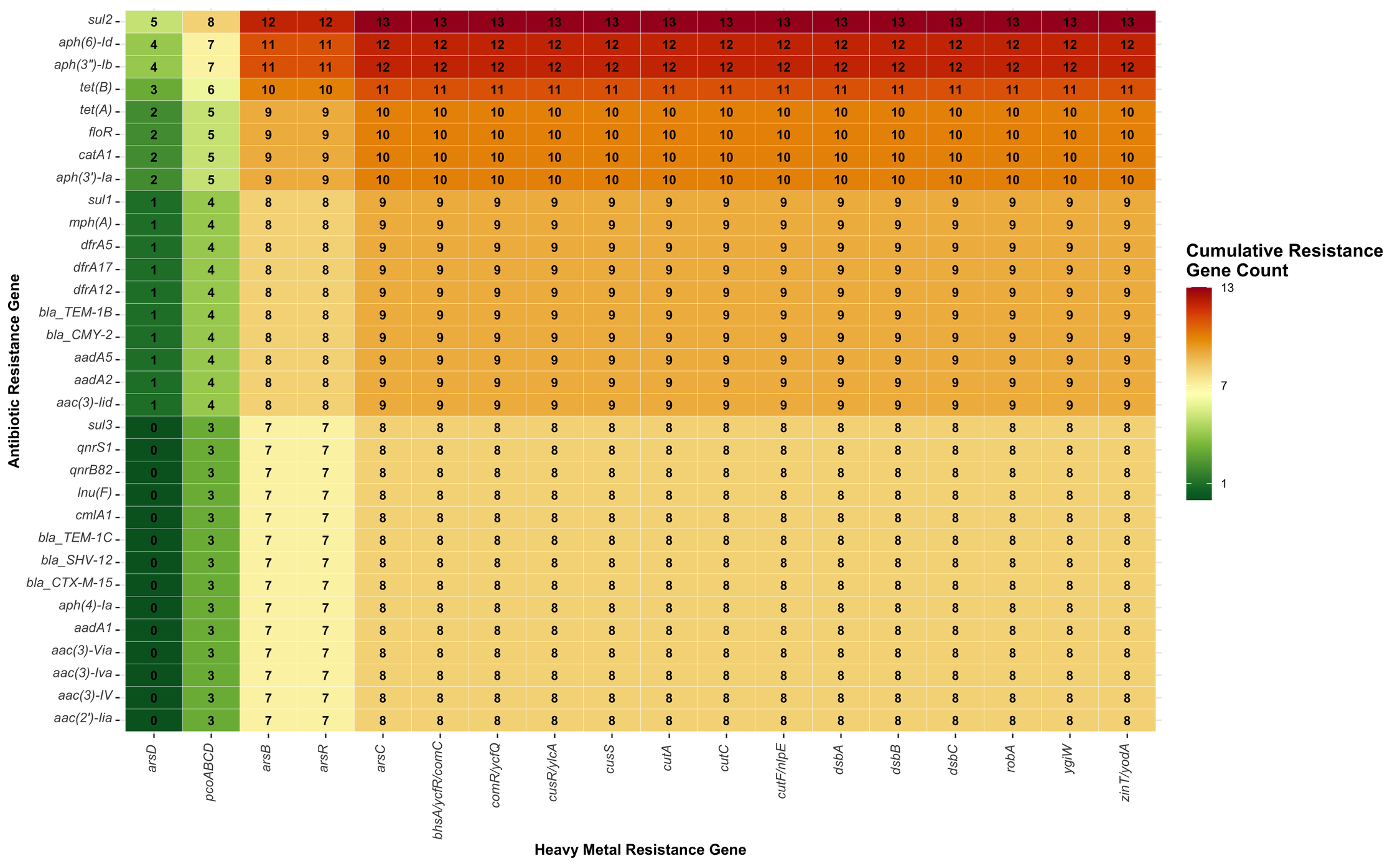

Supplement: S2 Fig — (TIF) [file pone.0294565.s005.tif]

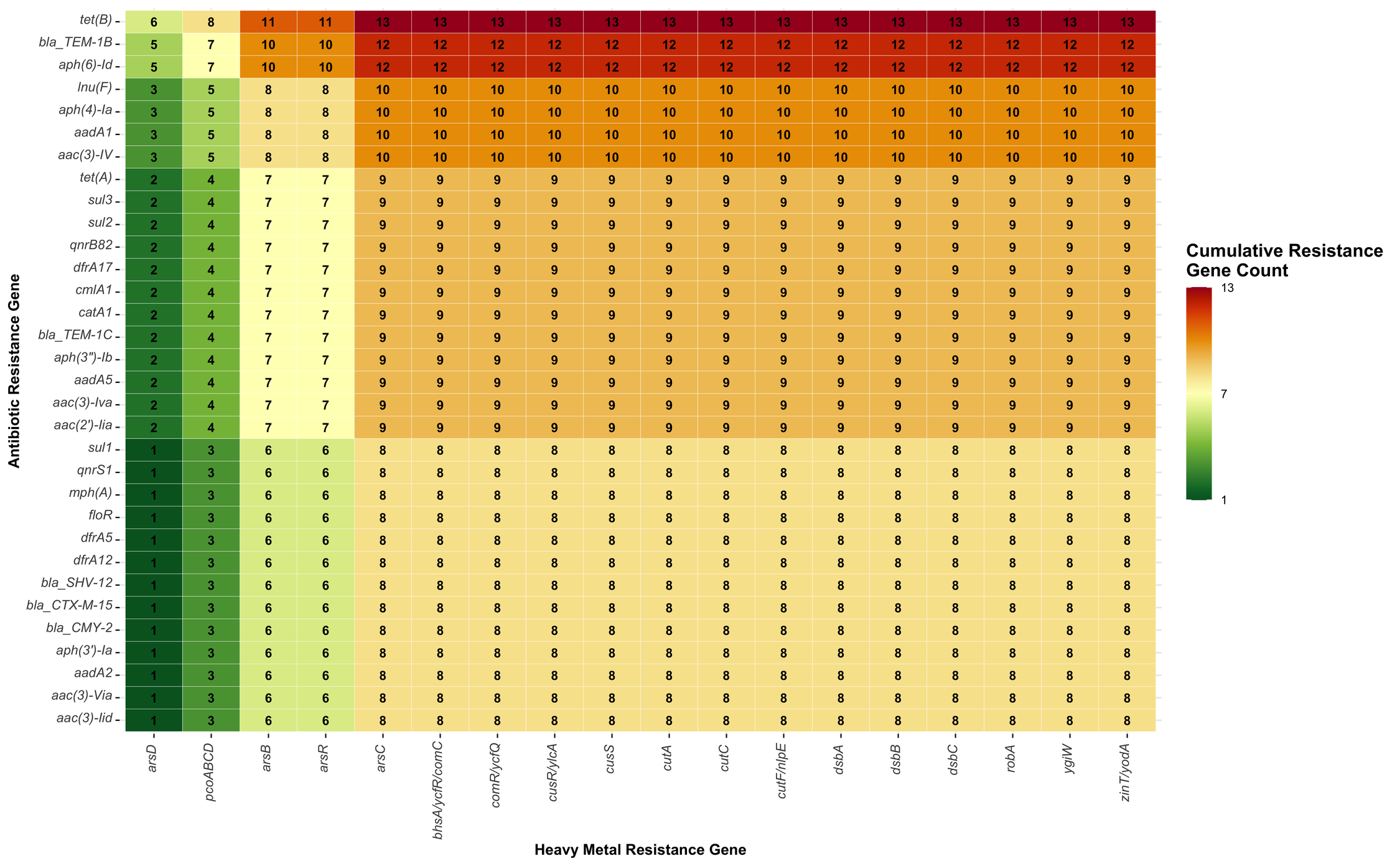

Supplement: S3 Fig — (TIF) [file pone.0294565.s006.tif]
