# Supplementary material for: Study of heavy metal resistance genes in Escherichia coli isolates from a marine ecosystem with a history of environmental pollution (arsenic, cadmium, copper, and mercury)
Source: PLoS One. 2023 Nov 16;18(11):e0294565. doi: 10.1371/journal.pone.0294565 (PMC10653420; doi:10.1371/journal.pone.0294565)
Supplement: S1 File — (DOCX) [file pone.0294565.s007.docx]

**Table of Contents**

[1. Results 2](#_Toc128578762)

[1.1. Geographic variation of heavy metal resistance genes in isolates from water sources 2](#_Toc128578763)

[1.2. Antibiotic resistance genes, heavy metal resistance genes, plasmids, and Superfund sites 3](#_Toc128578764)

[1.2.1. *E. coli* isolates from water sources 3](#_Toc128578765)

[1.2.2. *E. coli* isolates from marine mammal sources 4](#_Toc128578766)

# 1. Results

## 1.1. Geographic variation of heavy metal resistance genes in isolates from water sources

The *pcoABCD* gene system was found more often in isolates from marine mammals (three river otters and three harbor seals) than from water sources (only one from a Strait of Juan de Fuca sample) (S1 Table). The *zinT/yodA*, *cusR/ylcA*, and *cusS* genes were carried by all isolates from marine mammals in the study. Overall, for the seven non-universally found HMRGs, there did not appear to be a relationship between different regions of the Salish Sea and the carriage of HMRGs.

All isolates that carried expressed ARGs (n=25) carried *zinT/yodA*, *cusR/ylcA*, and *cusS* genes, 88% (n=22) carried *arsB* and *arsR* genes, 24% (n=6) carried *pcoABCD* genes, and 4% (n=1) carried *arsD* gene (Table 2). Among 276 isolates that did not carry any ARGs, 99% (n=274) carried *cusR/ylcA* and *cusS,* 99% (n=273) carried *zinT/yodA*, 74% (n=203) carried *arsB*, 73% (n=202) carried *arsR*, 4% (n=10) carried *arsD*, and 0.4% (n=1) carried *pcoABCD*. Qualitatively, we did not observe a difference in carriage of the seven non-universally found HMRGs between isolates that also carried ARGs versus isolates that did not carry any ARGs.

## 1.2. Antibiotic resistance genes, heavy metal resistance genes, plasmids, and Superfund sites

### 1.2.1. *E. coli* isolates from water sources

The two isolates from fresh water samples (GG 14-5 Cef and GG 14-6 Cef) had the *mph*(A) gene genetically linked with eight HMRGs (*cusR/ylcA, cusS, cutC, cutF/nlpE, dsbA, dsbC, robA,* and *zinT/yodA*; *comR/ycfQ, cusR/ylcA, cusS, cutC, cutF/nlpE, dsbC, robA,* and *zinT/yodA,* respectively). Additionally, the GG 14-5 Cef isolate was phenotypically resistant to aztreonam, cefotaxime, and cefepime and carried no plasmid sequences. The GG 14-6 Cef isolate was phenotypically resistant to numerous antibiotics (aztreonam, cefepime, cefotaxime, ceftazidime, ciprofloxacin, doxycycline, levofloxacin, minocycline, ticarcillin/clavulanic acid, and trimethoprim/sulfamethoxazole) and carried the IncFIA plasmid sequence, though there was no linkage with the resistance genes and the IncFIA plasmid or good correlation between phenotype and gene carriage (Table 2).

Both of the isolates from the Central Salish Sea that carried ARGs and were phenotypically resistant or intermediate were located near (i.e., directly within or geographically adjacent to) Superfund sites. One was sampled from Liberty Bay (344914-013-1036) located near the Naval Undersea Warfare Engineering Station, was phenotypically resistant to tetracycline and doxycycline, and carried 17 HMRGs (*arsB* [two copies], *arsC, arsR, bhsA/ycfR/comC, comR/ycfQ, cusR/ylcA, cusS, cutA, cutC, cutF/nlpE, dsbA, dsbB, dsbC, robA, ygiW,* and *zinT/yodA*), *aph(3")-Ib, aph(6)-Id,* and *tet*(B) ARGs, and the IncFIB(AP001918) plasmid sequence, though none of the HMRGs or ARGs were located on it. The other Central Salish Sea isolate (339940-002-477), sampled from Yukon Harbor, was located near the Old Navy Dump/Manchester Laboratory Superfund site, carried 17 HMRGs (*arsB*, *arsC, arsR, bhsA/ycfR/comC, comR/ycfQ, cusR/ylcA, cusS, cutA, cutC, cutF/nlpE* [two copies]*, dsbA, dsbB, dsbC, robA, ygiW,* and *zinT/yodA*), the *bla*_CMY-2_ ARG, and the IncI1-Iα plasmid, and phenotypically resistant to ﻿cefotaxime and ceftazidime. Neither of the Central Salish Sea isolates had genetic linkage between the HMRGs and ARGs in their genomes.

### 1.2.2. *E. coli* isolates from marine mammal sources

Another harbor seal, WDFW2019-154 (AN0107), was phenotypically resistant to amoxicillin, gentamicin, and trimethoprim/sulfamethoxazole and carried *aac(3)-Iid, aadA2, bla*_TEM-1B_*, dfrA12, mph*(A), and *sul1* ARGs, one copy of each of the 18 HMRGs except for *arsB, arsD, arsR,* and *pcoABCD*, and carried Col156 and IncFII(29) plasmid sequences. In this isolate, the *sul1* gene was located on the Col156 plasmid and nothing was associated with the IncFII(29) plasmid.

In two isolates from river otters in Black River (BR1E and BR1F), the position of aph(3'')-Ib, aac(3)-IV, and blaTEM-1B ARGs were linked upstream to 13 HMRGs (arsB, comR/ycfQ, cusR/ylcA, cusS, cutC, cutF/nlpE, dsbA, dsbC, pcoA, pcoB, pcoD, robA, and zinT/yodA). While both isolates also carried the IncI1-Iα plasmid sequence, none of the resistance genes were associated with the plasmid. The BR1E isolate was phenotypically resistant to doxycycline, gentamicin, and tobramycin, while the BR1F isolate was phenotypically resistant to ampicillin, gentamicin, tetracycline, and minocycline. Two other river otter isolates (CWG3J and HAM6D) in Cottonwood Grove Park carried HMRGs, ARGs, and plasmids, but none of the resistance genes were located on a plasmid or linked to each other (Table 2).
