# Supplementary material for: Study of heavy metal resistance genes in Escherichia coli isolates from a marine ecosystem with a history of environmental pollution (arsenic, cadmium, copper, and mercury)
Source: PLoS One. 2023 Nov 16;18(11):e0294565. doi: 10.1371/journal.pone.0294565 (PMC10653420; doi:10.1371/journal.pone.0294565)
Supplement: Attachment — Submitted filename: Comments to authors.docx [file pone.0294565.s008.docx]

**Comments to authors:**
The current study is very interesting; however, the authors should address the following comments to improve the quality of the manuscript and to make it looks more beneficial to human health :

1- Title:
   Please modify the title: All heavy metals mentioned in the title can be written between brackets in the end of the title, example: “study of heavy metal resistance genes in Escherichia coli isolates from a marine ecosystem with a history of environmental pollution, focusing on (arsenic, cadmium, copper, and mercury)”
2- Abstract:
   - Rephrase the main conclusion of your findings to sound better.
3- Introduction: (it needs to be more informative), try to provide some information about the relationship between the effects of heavy metals in E.Coli and human health.
   - Give a hint and some details about the spread of ARGs among and between humans and animals sources of HMRGs in bacterial environmental reservoirs.

- The authors should illustrate the importance of the study with regard to public health linked to the study objectives.
- Authors could add some paragraphs to fulfill the following gabs:

1- The relationship between the use of some metals as antibiotics for human treatment and antimicrobial resistance, heavy metal exposure, and resistance.
   -Rephrase the aim of the work to be clear and better sound.
2- Write some paragraphs about *Escherichia coli.*

   3- Material and methods
   - R-write the methodology according to the PLOs guidelines.

-The Ethical statement should be placed before the sampling section since there samples taken from animals marine mammals (harbor seals, harbor porpoises, river otters).
-Isolation techniques analysis of samples isolates, should be modified.

- Methods of fecal samples collection and laboratory tests should be clearly described.

- Methods for isolating genes must be written, even if they are performed by contracting with a laboratory not associated with the study, regardless of its reliability or reputation

-Results:
- Increase the resolution of the two figures to be 600 dpi.
- Place Fig 1. “Total count of heavy metal resistance genes per E. coli isolate” and Fig.2 “Total count of antibiotic resistance genes per E. coli isolate source “ in their right place (not in the appendix)

- The manuscript is written well but needs minor revision for the grammatical errors and spelling mistakes.
- Please revise the references with the correct style of the journal.
