# Supplementary material for: Study of heavy metal resistance genes in Escherichia coli isolates from a marine ecosystem with a history of environmental pollution (arsenic, cadmium, copper, and mercury)
Source: PLoS One. 2023 Nov 16;18(11):e0294565. doi: 10.1371/journal.pone.0294565 (PMC10653420; doi:10.1371/journal.pone.0294565)
Supplement: Attachment — Submitted filename: Response to Reviewers.docx [file pone.0294565.s009.docx]

Response to reviewer comments

PLOS ONE Manuscript Number: PONE-D-23-17575

Title: Carriage of arsenic, cadmium, copper, and mercury heavy metal resistance genes in *Escherichia coli* isolates from a marine ecosystem with a history of environmental pollution

| **Section** | **Reviewer Comment** | **Author Response** |
| --- | --- | --- |
| **Reviewer 1** | | |
| Overarching Feedback | The manuscript is based on impressive empirical evidence and makes an original contribution. Only minor revisions are needed before it can be published. | Thank you for your review of our manuscript and the thoughtful comments. In line with your comments, we have revised the manuscript for greater clarity and readability in certain sections. |
| Abstract | 6. Line no. 23 page no. 2- sentence should be read as "We screened 18 functional . . | We have updated this sentence and removed “for” (line 23). |
|  | 7. Line no. 24 page no. 2 sentence should be followed as "geographical variations . . | We have updated this sentence and corrected the phrase to “geographical variations” (line 24). |
| Introduction | 1. The authors conduct very relevant research, but unable to emphasis the relevance in the introduction. | We have modified the introduction to emphasize the public health relevance of our research (lines 49-76). |
| Materials and Methods | 2. To improve the readability of the paper, I suggest dividing the analysis into several subsections. | We agree with your suggestion. We have divided the analysis into multiple subsections to improve the readability of the paper (lines 102-253). |
| Discussion | 5. There should be one more section conclusion/summary and future prospective to better reflect their work. | Thank you for your suggestion. We added one paragraph at the end of the Discussion section to discuss conclusions and future directions of our work (lines 486-499). |
| Tables | 3. Table 2 is too long and difficult to understand, it should be adjusted. | We have updated Table 2 to be more comprehensible and have moved it to Supplementary Material (now Supplementary Table 2). |
| Figures | 4. Quality of figures should be improved, Text is very blurred and it is difficult to read black letters on dark green background. | We increased the resolution of all 5 figures (Fig 1, Fig 2, S1 Fig, S2 Fig, and S3 Fig) to be 600 dpi, and then uploaded each figure to the PACE tool to ensure they meet PLOS ONE figure guidelines. All figure uploads are the PACE-corrected figures. We have also bolded the text labels for greater legibility. |
| **Reviewer 2** | | |
| Overarching Feedback | The current study is very interesting; however, the authors should address the following comments to improve the quality of the manuscript and to make it looks more beneficial to human health : | Thank you for your review of our manuscript and the thoughtful comments. In line with your comments, we have revised the manuscript for greater clarity and phrasing of certain sections. |
| Title | Please modify the title: All heavy metals mentioned in the title can be written between brackets in the end of the title, example: “study of heavy metal resistance genes in Escherichia coli isolates from a marine ecosystem with a history of environmental pollution, focusing on (arsenic, cadmium, copper, and mercury)” | Thank you for your suggestion. We have updated both the full title and the short title (lines 1-4). |
| Abstract | Rephrase the main conclusion of your findings to sound better. | Thank you for your comment. We rephrased the main conclusion of our findings in the Abstract (lines 32-38). |
| Introduction | (it needs to be more informative), try to provide some information about the relationship between the effects of heavy metals in E.Coli and human health. | Thank you for your suggestion. We added more information about the relationship between effects of heavy metals in *E. coli* and human health in the Introduction section (lines 49-76). |
|  | Give a hint and some details about the spread of ARGs among and between humans and animals sources of HMRGs in bacterial environmental reservoirs | We added more details about spread of ARGs among and between human and animal sources of HMRGs in bacterial environmental reservoirs in the Introduction section (lines 49-61, 70-76). |
|  | The authors should illustrate the importance of the study with regard to public health linked to the study objectives. | We expanded upon the public health importance of our study linked to the study objectives in the Introduction section (lines 49-76). |
|  | Authors could add some paragraphs to fulfill the following gabs:   1- The relationship between the use of some metals as antibiotics for human treatment and antimicrobial resistance, heavy metal exposure, and resistance.  - Rephrase the aim of the work to be clear and better sound.  2- Write some paragraphs about Escherichia coli. | Thank you for your suggestions. We rephrased our study aim (lines 77-81) and added a few paragraphs about *E. coli* (lines 62-69) in the Introduction section. |
| Materials and Methods | R-write the methodology according to the PLOs guidelines. | We have re-written our “Materials and Methods” section based on the PLOS guidelines. |
|  | The Ethical statement should be placed before the sampling section since there samples taken from animals marine mammals (harbor seals, harbor porpoises, river otters). | We have added in an ethics statement before the sampling section (lines 92-101). |
|  | Isolation techniques analysis of samples isolates, should be modified. | We have added more detail about the isolation techniques of sample isolates (lines 102-132). |
|  | Methods of fecal samples collection and laboratory tests should be clearly described. | We have added more detail about the methods of fecal samples collection and laboratory tests (lines 102-132). |
|  | Methods for isolating genes must be written, even if they are performed by contracting with a laboratory not associated with the study, regardless of its reliability or reputation | We have added more detail about the methods for isolating genes (lines 117-132). |
| Results | Increase the resolution of the two figures to be 600 dpi. | We increased the resolution of all 5 figures (Fig 1, Fig 2, S1 Fig, S2 Fig, and S3 Fig) to be 600 dpi, and then uploaded each figure to the PACE tool to ensure they meet PLOS ONE figure guidelines. All figure uploads are the PACE-corrected figures. |
|  | Place Fig 1. “Total count of heavy metal resistance genes per E. coli isolate” and Fig.2 “Total count of antibiotic resistance genes per E. coli isolate source “ in their right place (not in the appendix) | Fig 1 and Fig 2 are currently located in the main manuscript in the correct places. There are three supplementary figures (S1 Fig, S2 Fig, and S3 Fig) included as part of the Supporting Information.  According to PLOS ONE guidelines, the figure captions should be inserted in the manuscript, but the figures themselves should be uploaded separately. Please see: <https://journals.plos.org/plosone/s/submission-guidelines#loc-figures-and-tables> |
|  | The manuscript is written well but needs minor revision for the grammatical errors and spelling mistakes. | Thank you for your comment. We have gone through the manuscript and corrected any grammatical errors or spelling mistakes based on your suggestions. |
|  | Please revise the references with the correct style of the journal. | We have revised the references with the correct style of the journal (“Vancouver”). |
